# Efficacy of Long-Acting Injectable Cabotegravir and Rilpivirine Compared With Daily Oral Antiretroviral Therapy in Patients With HIV: A Systematic Review

**DOI:** 10.7759/cureus.103429

**Published:** 2026-02-11

**Authors:** Jesus Endara-Mina, Mayuri Quishpe, Emely Vera, Nicolás Haro, Alisson Guzmán, Jefferson Guillin, Kleber Quisnancela, Henry Sarmiento-Vallejo, Isabel Mantilla-Alcivar, Carlos Cabrera, Jairo Cueva, Shirley Serrano, Catherine Caiza, Cesar Intriago, Paulina Salazar

**Affiliations:** 1 Faculty of Legal and Political Sciences, Universidad Técnica Particular de Loja (UTPL), Loja, ECU; 2 Teaching and Research Unit, General Provincial Hospital Pablo Arturo Suárez, Quito, ECU; 3 Faculty of Public Health, Escuela Superior Politécnica de Chimborazo (ESPOCH), Riobamba, ECU; 4 Obstetrics and Gynecology, University of the Americas, Quito, ECU; 5 Faculty of Medical Sciences, Universidad Central del Ecuador (UCE), Quito, ECU; 6 Faculty of Medicine, University of the Americas, Quito, ECU; 7 Faculty of Chemical and Health Sciences, Universidad Técnica de Machala (UTMACH), Machala, ECU; 8 Research and Academic Affairs, Larkin Community Hospital, South Miami, USA; 9 Obstetrics and Gynecology, Hospital de los Valles, Quito, ECU

**Keywords:** adherence, cabotegravir, hiv, long-acting therapy, rilpivirine

## Abstract

Long-acting antiretroviral therapy (ART) has emerged as an innovative strategy to address limitations associated with daily oral regimens in people living with human immunodeficiency virus type 1 (HIV-1), including adherence barriers, treatment fatigue, and social stigma. This systematic review, conducted in accordance with Preferred Reporting Items for Systematic Reviews and Meta-Analyses (PRISMA) 2020 guidelines and registered in International Prospective Register of Systematic Reviews (PROSPERO, CRD420251155009), identified randomized clinical trials comparing long-acting injectable cabotegravir and rilpivirine (CAB/RPV-LA) with standard daily oral ART through searches of PubMed, ScienceDirect, the Cochrane Library, and Google Scholar. Ten randomized trials involving 5,619 adults with HIV-1 were included. CAB/RPV-LA, administered intramuscularly every four or eight weeks, achieved durable virological suppression exceeding 90% across follow-up periods of 48 to 240 weeks, demonstrating non-inferiority to daily oral ART. Patient-reported outcomes consistently favored the injectable regimen, with higher treatment satisfaction scores and excellent adherence within the dosing window, while adverse events were mainly mild-to-moderate injection-site reactions (ISRs), resulting in treatment discontinuation in fewer than 1% of participants. Overall, the certainty of evidence was high for virological efficacy and moderate for safety and satisfaction outcomes, supporting long-acting CAB/RPV-LA as an effective and well-tolerated alternative that improves adherence, convenience, and quality of life in long-term HIV management.

## Introduction and background

The human immunodeficiency virus (HIV) is a chronic viral infection characterized by persistent replication within CD4+ lymphocytes, which play a central role in coordinating the host immune response [1]. By integrating its genetic material into the host genome, HIV progressively depletes CD4+ T helper lymphocytes, resulting in immunosuppression and increased susceptibility to opportunistic infections and malignancies [2]. In the absence of effective treatment, sustained CD4+ cell decline leads to acquired immunodeficiency syndrome (AIDS), a condition associated with substantially increased morbidity and mortality [3].

Despite major advances in prevention and treatment, HIV remains a significant global public health challenge. According to the Joint United Nations Programme on HIV/AIDS, approximately 39.9 million people were living with HIV worldwide by the end of 2023. In Ecuador, 48,782 confirmed cases had been reported by the same year, with an average of 14 new diagnoses per day, predominantly among men aged 25 to 49 years [4]. While regional epidemiological patterns vary, these figures underscore the persistent burden of HIV and the need for long-term, sustainable treatment strategies adaptable to diverse healthcare settings [5,6].

The introduction of combination antiretroviral therapy (ART) has fundamentally transformed the prognosis of HIV infection by enabling durable viral suppression, immune restoration, and a marked reduction in HIV-related mortality [7]. Clinically, viral suppression, commonly defined as plasma HIV RNA levels below 50 copies/mL, indicates effective control of viral replication, prevention of disease progression, and elimination of sexual HIV transmission risk. However, standard daily oral ART regimens, which require strict and lifelong adherence, continue to pose challenges. Treatment fatigue, suboptimal adherence, social stigma, and cumulative drug-related adverse effects may compromise long-term treatment success in some individuals [8,9].

In response to these limitations, pharmacological innovation has increasingly focused on long-acting injectable formulations designed to reduce pill burden, simplify treatment delivery, and improve patient experience. Among these, the long-acting injectable cabotegravir and rilpivirine (CAB/RPV-LA) has emerged as a novel therapeutic option. This regimen consists of cabotegravir, a long-acting integrase strand transfer inhibitor (INI-LA), and rilpivirine, a non-nucleoside reverse transcriptase inhibitor (NNRTI), administered intramuscularly every four or eight weeks [10]. Importantly, CAB/RPV-LA is currently indicated primarily as a maintenance therapy for individuals who are already virologically suppressed on oral ART and meet specific eligibility criteria, including the absence of resistance to either agent and the ability to attend scheduled injection visits.

The clinical efficacy and safety of CAB/RPV-LA have been evaluated in several multicenter phase III trials, including FLAIR (First Long-Acting Injectable Regimen), ATLAS (Antiretroviral Therapy as Long-Acting Suppression), and ATLAS-2M (Antiretroviral Therapy as Long-Acting Suppression - Every Two Months). These studies demonstrated virological non-inferiority compared with daily oral ART in selected populations, along with higher patient satisfaction, lower discontinuation rates, and a favorable safety profile [11,12]. Together, these findings suggest that long-acting injectable therapy may represent a meaningful shift toward more patient-centered HIV care, provided that practical considerations such as injection scheduling, healthcare access, and patient eligibility are carefully addressed.

The purpose of this systematic review is to comprehensively assess the clinical efficacy of the CAB/RPV-LA regimen compared with daily oral ART in people living with HIV infection. Primary outcomes include sustained virological suppression (HIV RNA <50 copies/mL), incidence of confirmed virological failure, treatment adherence, perceived acceptability, reported adverse events, and health-related quality of life.

## Review

Materials and methods

This systematic review was conducted following the Preferred Reporting Items for Systematic Reviews and Meta-Analyses (PRISMA) 2020 guidelines [13]. The study was registered retrospectively in the International Prospective Register of Systematic Reviews (PROSPERO) under the identifier CRD420251155009.

Search Strategy

A comprehensive literature search was performed across PubMed/Medical Literature Analysis and Retrieval System Online (MEDLINE), ScienceDirect, Cochrane Library, and Google Scholar to identify relevant studies. The search strategy combined Medical Subject Headings (MeSH) terms, free-text keywords, and Boolean operators to ensure exhaustive retrieval of eligible studies. The main search concepts included HIV, ART, cabotegravir, rilpivirine, and efficacy. No restrictions were applied concerning language, publication date, or geographical region. Additionally, manual searches were conducted through the reference lists of included trials to identify additional relevant records. Duplicates were removed prior to screening. Full search strings are provided in Table 1.

**Table 1:** Search strategy MeSH: medical subject headings; HIV: human immunodeficiency virus; HIV-1: human immunodeficiency virus type 1; ART: antiretroviral therapy; CAB-LA: long-acting cabotegravir; RPV-LA: long-acting rilpivirine; RCT: randomized controlled trial

Concept	MeSH terms	Free terms/Natural language	Boolean combination
HIV / HIV-1 Infection	"HIV"[MeSH] OR "HIV Infections"[MeSH]	HIV OR HIV-1 OR HIV-1 infection OR human immunodeficiency virus	(HIV OR “HIV-1” OR “HIV-1 infection”)
Cabotegravir (Long-acting)	No specific MeSH term available	cabotegravir OR long-acting cabotegravir OR CAB-LA OR injectable cabotegravir	(cabotegravir OR “long-acting cabotegravir”)
Rilpivirine (Long-acting)	"Rilpivirine"[MeSH]	rilpivirine OR long-acting rilpivirine OR RPV-LA OR injectable rilpivirine	(rilpivirine OR “long-acting rilpivirine”)
Antiretroviral Therapy/Efficacy	"Anti-HIV Agents"[MeSH] OR "Antiretroviral Therapy, Highly Active"[MeSH]	antiretroviral therapy OR ART OR HIV treatment OR treatment efficacy OR virological suppression	(“antiretroviral therapy” OR ART OR “treatment efficacy” OR “virological suppression”)
Study Design (Trials)	"Randomized Controlled Trial"[Publication Type] OR "Clinical Trial"[Publication Type]	randomized controlled trial OR clinical trial OR randomized study OR RCT OR controlled trial	(“randomized controlled trial” OR “clinical trial” OR “randomized study”)

Inclusion and Exclusion Criteria

Study eligibility and outcome selection were defined using the Participants, Intervention, Comparison, and Outcome (PICO) framework. Eligible studies included adults and adolescents with confirmed HIV-1 infection receiving the CAB/RPV-LA compared with standard daily oral ART. The primary outcome of interest was virological efficacy, defined as the proportion of participants achieving or maintaining plasma HIV RNA levels below 50 copies/mL. Secondary outcomes included safety and tolerability profiles, incidence of adverse events, and measures of treatment adherence and patient satisfaction.

Randomized controlled trials (RCTs) evaluating CAB/RPV-LA versus standard daily oral ART were eligible for inclusion. Both open-label and blinded trials were considered, provided they employed a prospective randomized design, reported primary virological efficacy outcomes, and had a minimum follow-up duration of three months. Studies were excluded if they: included vulnerable populations such as children under 12 years of age, pregnant women, or individuals with significant co-infections (e.g., hepatitis B or tuberculosis); did not clearly specify the intervention or comparator protocols; or were non-randomized studies, observational designs, reviews, editorials, commentaries, or correspondence articles.

Study Selection

Titles and abstracts were independently screened by two reviewers (MQ and EV) to determine eligibility. In cases of uncertainty, full-text articles were retrieved for further assessment. Discrepancies were resolved by consensus or, when necessary, through arbitration by a third reviewer (JEM).

Data Extraction and Management

Data extraction was conducted using a standardized form adapted from the Cochrane Consumers and Communication Review Group. Extracted variables included the author, year of publication, country, total sample size, follow-up duration, intervention details, measured variables, and main outcomes. Two reviewers (NH and AG) independently extracted the data, and any disagreements were resolved through discussion or consultation with a third reviewer (CI).

Data Analysis and Synthesis

Due to the clinical and methodological heterogeneity of the included randomized trials, particularly regarding study phase, follow-up duration, dosing intervals, and population characteristics, a meta-analysis was not feasible. Instead, a structured qualitative synthesis was performed. The findings were summarized and compared according to study design, sample size, intervention regimen, follow-up period, and primary outcomes. This approach enabled an integrated assessment of virological efficacy, safety and tolerability, treatment adherence, and patient-reported satisfaction across trials.

Methodological Quality Assessment

The methodological quality of the included trials was assessed using the Jadad scale [14] and the Grading of Recommendations, Assessment, Development and Evaluation (GRADE) system [15] to determine the certainty of evidence. Only studies scoring ≥3 on the Jadad scale and rated as having at least moderate certainty according to GRADE were included in the final synthesis. Risk of bias was evaluated according to GRADE domains: selection bias, performance bias, detection bias, attrition bias, and reporting bias. In addition, the Cochrane Risk of Bias 2.0 (RoB 2) was employed to provide a structured appraisal of methodological rigor [16]. To ensure transparency and reproducibility, a PRISMA 2020-based checklist was applied throughout all stages of the review process.

Results

**Figure 1:**
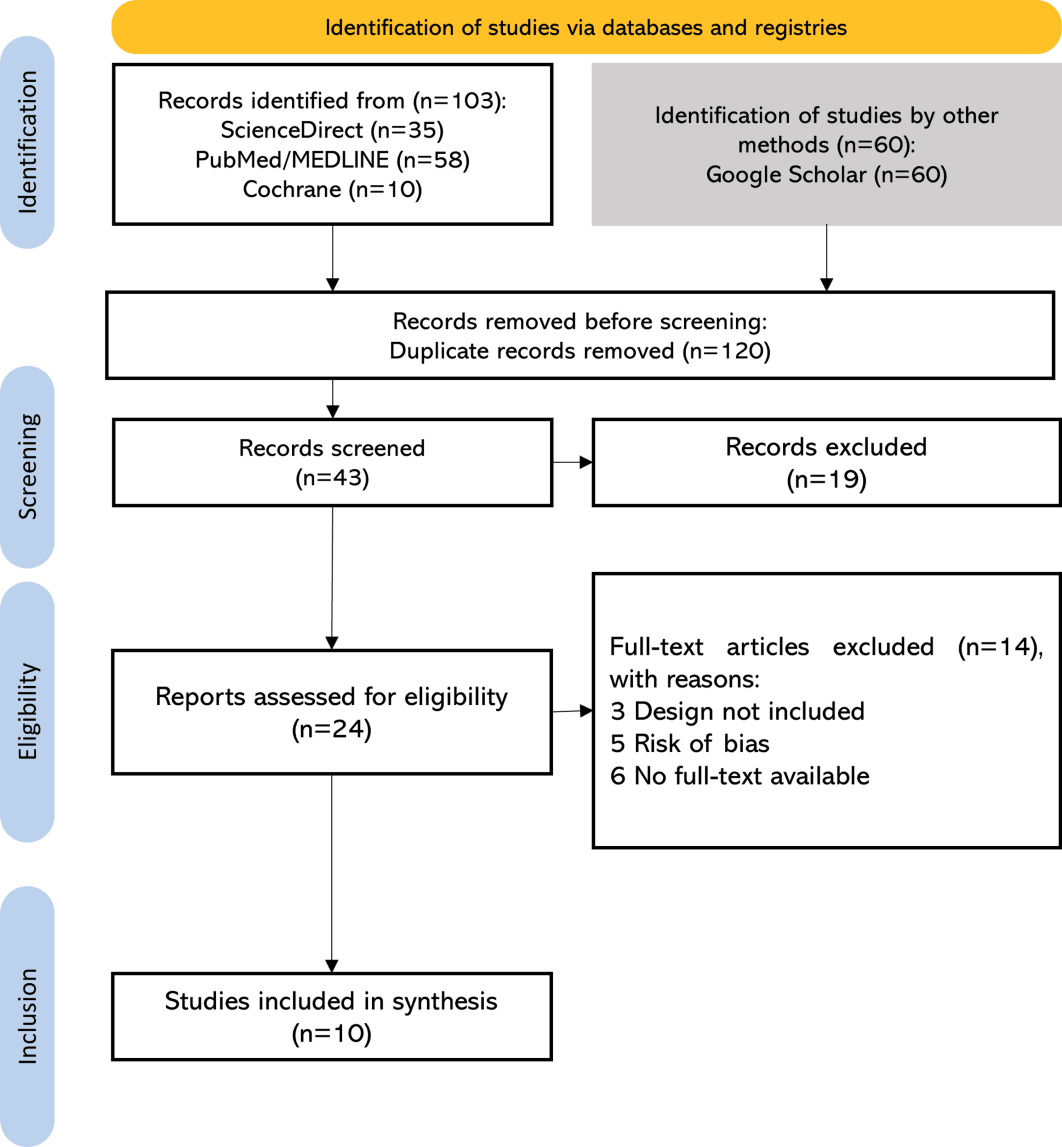
PRISMA flowchart for the identification and selection of included studies PRISMA: Preferred Reporting Items for Systematic Reviews and Meta-Analyses; MEDLINE: Medical Literature Analysis and Retrieval System Online

Through the application of predefined search strategies and eligibility criteria, a total of 163 records were initially identified. After removing 120 duplicates, 43 unique records were screened by title and abstract. Of these, 10 were excluded based on study design, and nine were excluded due to incomplete full-text access. A total of 24 articles were assessed in full, of which 10 RCTs fulfilled the inclusion criteria. Additionally, five articles were excluded during the full-text screening phase due to a high risk of selection bias, as determined by the RoB 2. The study selection process is summarized in Figure 1.

Characteristics of the Included Studies

Table 2 summarizes the general characteristics of the included randomized clinical trials, categorized by geographic region, methodological design, study population, treatment regimen, and primary outcomes. Collectively, the 10 studies correspond to phase 2b-3b clinical trials, predominantly open-label and multicenter in design. A smaller subset consisted of extension or implementation studies conducted in real-world clinical settings, all maintaining prospective protocols and non-inferiority frameworks compared with standard daily oral ART.

**Table 2:** Characteristics of the included randomized clinical trials ^1 ^Note: The total number of studies in each category does not always equal the total number of trials (n = 10) because several studies evaluated more than one intervention schedule (e.g., monthly and bi-monthly dosing) or reported multiple outcome domains (e.g., efficacy, safety, and patient-reported satisfaction). Subcategories are therefore not mutually exclusive. HIV: human immunodeficiency virus; RNA: ribonucleic acid; ART: antiretroviral therapy

Category	Subcategory	Number of studies (n)
Geographical region	North America and Europe	3
	Africa	1
	Oceania	1
	Multinational (≥3 continents)	5
Study design	Randomized, double-blind phase 2b	2
	Randomized, open-label phase 3 – 4	8
Sex distribution	Predominantly male (>70%)	7
	Balanced (≈50% male/female)	1
	Moderate female representation (30–40%)	2
Treatment regimen ^1^	Monthly injections	5
	Bi-monthly injections	4
	Oral induction before injection	6
Follow-up duration	48 weeks	6
	96 weeks	2
	≥144 weeks (long-term extension)	2
Outcomes ^1^	Virological suppression (HIV RNA <50 copies/mL)	10
	Safety and tolerability	10
	Patient satisfaction/preference	5
	Pharmacokinetics/dosing optimization	2
Overall efficacy	Non-inferior to oral ART	9
	Maintained suppression after long-term use	1
Geographic diversity of participants	Multicontinental cohorts including low- and middle-income countries	4
	High-income settings only	6

The analyzed populations primarily included adults with stable virological suppression, followed by ART-naïve individuals and a smaller group of adolescents. Male participants represented the majority (70-80%), though recent studies expanded inclusion to women and to participants from African and middle-income regions. Follow-up durations ranged from 48 to 240 weeks, allowing for a comprehensive assessment of both sustained efficacy and long-term safety of long-acting injectable regimens.

Regarding treatment regimens, most studies compared monthly (Q4W) and bimonthly (Q8W) intramuscular administration of cabotegravir plus rilpivirine, both demonstrating viral suppression rates exceeding 90% and favorable tolerability. Primary outcomes focused on virological efficacy and safety, while secondary analyses included pharmacokinetic parameters and patient satisfaction assessments.

Critical Appraisal

The methodological quality assessment indicated that the included RCTs exhibited a generally high level of scientific rigor. According to the Jadad scale, most trials scored between 3 and 5 points, reflecting adequate reporting of randomization, handling of withdrawals, and coherent application of blinding procedures where feasible. Although open-label designs presented inherent limitations, particularly the inability to blind participants or investigators, methodological robustness was maintained through strong bias control and consistency of primary outcomes.

The certainty of evidence, evaluated through the GRADE framework, was rated as high for virological efficacy and moderate for safety and patient satisfaction outcomes, mainly due to variability in adverse event reporting and subjective adherence measures. During full-text screening, five studies were excluded for high selection bias, as determined by the Cochrane RoB 2 tool (Table 3).

**Table 3:** Risk of bias in studies GRADE: Grading of Recommendations Assessment, Development and Evaluation; JADAD: Jadad Scale (Oxford Quality Scoring System)

Study (Author, Year)	Bias from the randomization process	Bias due to intended interventions	Bias due to missing outcome data	Bias in the measurement of the outcomes	Bias in the selection of the reported result	Overall risk of bias	Level of evidence (GRADE)/Level of quality (JADAD)
Margolis DA et al., 2015 [17]	Some concerns	Some concerns	Low risk	Low risk	Some concerns	Some concerns	Moderate (limited by partial blinding and phase 2b design) 3/5 (adequate quality); open-label study without full blinding
Margolis DA et al., 2017 [18]	Low risk	Some concerns	Low risk	Low risk	Some concerns	Low risk	High 4/5 (Adequate quality), no losses reported
Orkin C et al., 2020 [19]	Low risk	Some concerns	Low risk	Low risk	Some concerns	Low risk	High 3/5 (adequate quality), incomplete blinding
Swindells S et al., 2020 [20]	Low risk	Some concerns	Low risk	Low risk	Some concerns	Low risk	High 3/5 (adequate quality), not fully blinded
Overton ET et al., 2023 [21]	Low risk	Some concerns	Low risk	Low risk	Some concerns	Low risk	High 3/5 (adequate quality); open-label design without full blinding
Kityo C et al., 2024 [22]	Low risk	Some concerns	Low risk	Low risk	Some concerns	Low risk	High 3/5 (adequate quality), not complete blindness
Jonsson-Oldenbüttel C et al., 2024 [23]	Low risk	Some concerns	Low risk	Low risk	Some concerns	Low risk	Moderate (high applicability, no direct control group), 3/5 (adequate quality), no blinding, withdrawals reported
Mills A et al., 2022 [24]	Some concerns	Some concerns	Low risk	Low risk	Some concerns	Some concerns	Moderately controlled data, but with limitations 3/5 (adequate quality), incomplete blinding
Gaur AH et al., 2024 [25]	Some concerns	Some concerns	Low risk	Low risk	Some concerns	Some concerns	High 3/5 (adequate quality), no complete blinding
John M et al., 2024 [26]	Some concerns	Some concerns	Low risk	Low risk	Some concerns	Some concerns	Moderate (indirect effect estimation; no control group, but well-conducted, with high applicability to real-world settings) 3/5 (adequate quality, no blinding)

Synthesis of Results

The 10 RCTs included in this systematic review were conducted between 2015 and 2024, encompassing a total of 5,619 participants with HIV-1 infection, of whom 2,068 received long-acting cabotegravir/rilpivirine injections. The trials were conducted across multiple countries, including the United States, Canada, Spain, France, Germany, South Africa, Mexico, Uganda, and Japan. Most studies were multinational, with the highest representation from the U.S. and Europe. The complete information from the studies is summarized in Table 4. 

**Table 4:** Summary of studies CAB/RPV-LA: long-acting injectable cabotegravir and rilpivirine; ART: antiretroviral therapy; HIV-1: human immunodeficiency virus type 1; Q4W: every four weeks; Q8W: every eight weeks; ISR: injection-site reaction; PK: pharmacokinetics; RCT: randomized controlled trial; NNRTI: non-nucleoside reverse transcriptase inhibitor; INI: integrase inhibitor; EFV: efavirenz; DTG: dolutegravir; ABC: abacavir; 3TC: lamivudine; I-STI: integrase strand transfer inhibitor; ISRs; injection site reactions; NRTIs: nucleoside reverse transcriptase inhibitors; PI: protease inhibitor; RPV: rilpivirine, non-nucleoside reverse transcriptase inhibitor; WHO: World Health Organization

Author/Year	Phase	Population (n)/Follow-up	Intervention/Comparator	Primary outcome	Key results	Conclusion
Margolis DA et al., 2015 [17]	Randomized, phase 2b, dose-ranging study	Sample: 243 patients- cabotegravir + rilpivirine: 122 patients -efavirenz + NRTIs: 121 patients, Follow-up: 96 weeks.	Cabotegravir (10/30/60 mg) + NRTIs during induction, followed by cabotegravir + rilpivirina. Control: Efavirenz + NRTIs.	Virological suppression (<50 copies/mL)	86% vs 83% maintained suppression; good tolerability	Long-acting regimen viable post oral induction with comparable efficacy to EFV.
Margolis DA et al., 2017 [18]	Phase 2b, randomized, open-label, multicenter, non-inferiority clinical trial	Sample: 595 patients - cabotegravir and rilpivirine: 309 patients. -cabotegravir and rilpivirine: 309 patients. Follow-up: 96 weeks.	Group 1: Cabotegravir LA 400 mg + rilpivirina LA 600 mg IM every 4 weeks. Group 2: Cabotegravir LA 600 mg + rilpivirina LA 900 mg IM every 8 weeks. Group 3: Cabotegravir 30 mg + abacavir/lamivudine oral daily.	Viral suppression rate	≥94% in both arms; mild ISRs	Confirmed durable efficacy; Q8W non-inferior to Q4W.
Orkin C et al., 2020 [19]	Phase 3, multicenter, randomized, open-label, non-inferiority clinical trial	Sample: 566 patients - cabotegravir and rilpivirina: 283 patients. - maintenance antiretroviral therapy: 283 patients. Follow-up: 48 weeks.	Group 1: Monthly injectable cabotegravir + rilpivirine (after oral induction). Group 2: Daily oral dolutegravir–abacavir–lamivudine.	HIV RNA <50 copies/mL	93.6% vs 93.3% suppression; high satisfaction	Non-inferior efficacy; higher patient satisfaction.
Swindells S et al., 2020 [20]	Phase 3, randomized, open-label, multicenter, non-inferiority trial	Sample: 616 patients - cabotegravir plus rilpivirine: 308 patients. - maintenance antiretroviral therapy: 308 patients. Follow-up: 48 weeks.	Group 1: Monthly injectable therapy: Cabotegravir LA + Rilpivirine LA (IM). Group 2: Continuous standard oral therapy (2 NRTIs +PI/I-STI/NNRTI).	Virological response	92.5% vs 95.5% maintained suppression	Monthly injections are non-inferior to daily ART.
Overton ET et al., 2023 [21]	Phase 3b, randomized, open-label, multicenter, non-inferiority clinical trial	Sample: 1.045 patients - cabotegravir and rilpivirine: 523 patients. -maintenance antiretroviral therapy: 522 patients. Follow-up: 48 weeks.	Group 1: Cabotegravir 600 mg + rilpivirine 900 mg IM every 8 weeks. Group 2: Cabotegravir 400 mg + rilpivirina 600 mg every 4 weeks (comparator group).	Virological non-inferiority	94% vs 93%; ISRs mild; low discontinuation	Bi-monthly dosing is equally effective and well-tolerated.
Gaur A et al., 2024 [25]	Phase 1/2, open-label, non-comparative, multicenter clinical trial.	Sample: 55 patients - cabotegravir (CAB) - cohort 1Ca: 30 patients. Rilpivirine (RPV) - Cohort 1R: 25 patients. Follow-up: 16 weeks (acute phase) - 48 weeks (prolonged pharmacokinetics).	Group 1: Oral cabotegravir 30 mg/day for 4–6 weeks + long-acting IM cabotegravir every 4 or 8 weeks. Group 2: Oral rilpivirine 25 mg/day for 4–6 weeks + long-acting IM rilpivirine every 4 or 8 weeks.	PK and safety	Plasma exposure consistent with adults; full suppression	Supports dosing extrapolation for adolescents.
Mills et al., 2022 [24]	Randomized, multicenter, open-label, phase 2b clinical trial	Sample: 97 patients - CAB+RPV long-acting Q2M (Long-acting every 2 months): 90 patients. - Oral DTG/RPV antiretroviral therapy: 7 patients. Follow-up: 48 weeks.	Group 1: CAB+RPV intramuscular every 2 months (600 mg + 900 mg); Group 2: Dolutegravir 50 mg + rilpivirina 25 mg oral daily.	Real-world efficacy	>90% suppression; high adherence	Confirms feasibility in resource-limited settings.
Jonsson-Oldenbüttel C et al., 2024 [23]	Phase 3b, multicenter, hybrid type III implementation-effectiveness, open-label clinical trial	Sample: 430 patients - cabotegravir 600 mg + rilpivirina 900 mg IM every 2 months: 215 patients. - Oral induction in 1 month: 215 patients. Follow-up: 48 weeks.	Group 1: Cabotegravir 600 mg + Rilpivirina 900 mg IM Group 2: Conventional antiviral treatment oral induction within 1 month.	Effectiveness and satisfaction	91.8% suppression; improved adherence	Demonstrates real-world applicability.
John M et al., 2024 [26]	Phase 4, single-center, open-label, single-arm, prospective clinical trial using mixed methods	Sample: 60 patients - cabotegravir 600 mg + rilpivirina 900 mg IM every 2 months: 30 patients -oral induction in 1 month: 30 patients. Follow-up: 48 weeks.	Group 1: Cabotegravir 600 mg + rilpivirina 900 mg IM every 2 months. Group 2: Tenofovir + lamivudina + dolutegravir oral daily (WHO standard regimen).	Adherence and feasibility	97.2% on-time dosing; good tolerance	Excellent adherence; supports integration into clinical practice.
Kityo C et al., 2024 [22]	Randomized, multicenter, open-label, non-inferiority, phase 3 clinical trial	Sample: 512 patients - CAB+RPV IM: 255 patients -antiretroviral therapy tenofovir + lamivudine + Dolutegravir oral daily: 257 patients. Follow-up: 48 weeks.	Group 1: Cabotegravir 600 mg + Rilpivirina 900 mg IM every 8 weeks. Group 2: Tenofovir + lamivudina + dolutegravir oral daily (WHO standard regimen).	Virological suppression	93% maintained viral control	Validates regimen’s global applicability and safety.

Virological Suppression and Treatment Efficacy

Across the 10 randomized clinical trials included in this review, long-acting intramuscular cabotegravir and rilpivirine demonstrated high efficacy in maintaining HIV-1 virological suppression. Initial evidence from the LATTE (Long-Acting Antiretroviral Treatment Enabling) phase 2b trial [17] established the viability of this dual regimen following oral induction, with viral suppression rates exceeding 85% at week 96. The subsequent LATTE-2 trial [18] confirmed durable efficacy at week 96, demonstrating non-inferiority compared with daily oral ART. In large phase 3 trials such as FLAIR [19] and ATLAS [20], virological suppression was sustained in more than 90% of participants through 48 and 96 weeks, with outcomes comparable to standard oral ART.

The ATLAS-2M study [21] evaluated different dosing intervals within the long-acting regimen and reported that administration every eight weeks was non-inferior to monthly dosing in terms of virological suppression.

Safety and Tolerability

The overall safety profile of the injectable combination was favorable, with most adverse events being mild to moderate injection-site reactions (ISRs). LATTE-2 reported that 97% of participants experienced ISRs, though less than 1% discontinued therapy as a result [18]. Comparable safety outcomes were described in ATLAS, FLAIR, and ATLAS-2M, where pain and nodules were transient and diminished over time [20,21]. Data from real-world and implementation studies, including CARES (Cabotegravir and Rilpivirine Long-Acting in Africa) [22] and the European Implementation Study [23], reinforced these findings, indicating no new safety signals and high overall tolerability even in resource-limited settings.

Adherence, Satisfaction, and Quality of Life

Patient-reported outcomes consistently favored the long-acting regimen. In the FLAIR trial, treatment satisfaction increased after switching to injectable therapy, with 91% of participants expressing a preference for CAB/RPV-LA over daily oral ART at week 48 [19]. In ATLAS, patient-reported outcome measures demonstrated higher treatment satisfaction scores with injectable therapy compared with continued oral regimens, although preference was not quantified as a single aggregate percentage [20].

The ATLAS-2M trial further evaluated patient-reported adherence perceptions by comparing bi-monthly (every eight weeks) with monthly (every four weeks) administration of CAB/RPV-LA, showing no loss of perceived adherence with the extended dosing interval [21]. Long-term follow-up from Mills et al. indicated sustained preference for the injectable approach after more than five years of oral ART [24]. In African cohorts, the switch to long-acting formulations was associated with enhanced treatment satisfaction and fewer adherence barriers, reflecting the importance of regimen convenience in real-world contexts [22].

Pharmacokinetics and Dosing Interval

Pharmacokinetic analyses demonstrated that plasma concentrations of cabotegravir and rilpivirine remained above inhibitory thresholds throughout both every-four-week and every-eight-week dosing intervals. The phase 1/2 MOCHA (More Options for Children and Adolescents) trial in adolescents further showed pharmacokinetic exposure comparable to adult populations, supporting dose extrapolation [25]. Bimonthly dosing maintained adequate drug exposure without compromising viral suppression, as demonstrated by Overton et al., paving the way for flexible long-acting ART schedules [21].

Implementation and Real-World Effectiveness

Recent implementation trials provided complementary evidence of the regimen’s feasibility outside controlled settings. The European Implementation Study [23] and the Injectable Antiretroviral Feasibility Study (JABS) feasibility study [26] demonstrated real-world effectiveness exceeding 90% at 12 months across heterogeneous patient populations, including individuals with different prior ART histories, baseline viral suppression status, and sociodemographic backgrounds. Importantly, African data from the CARES trial offered the first large-scale evaluation in sub-Saharan settings, confirming non-inferiority to oral regimens and demonstrating practical applicability in resource-limited clinics [22].

Discussion

This systematic review, synthesizing evidence from 10 RCTs encompassing 5,619 adults with HIV-1 infection, provides a comprehensive evaluation of the efficacy, safety, adherence, and patient satisfaction associated with the CAB/RPV-LA. In the ATLAS-2M trial, Overton et al. randomized 1,045 virologically suppressed adults to receive CAB/RPV-LA either every four or eight weeks for 48 weeks [21]. Virological suppression (<50 copies/mL) was maintained in 94% of participants in the eight-week arm and 93% in the four-week arm, confirming non-inferiority (adjusted difference: 0.8%; 95% confidence interval (CI): -0.6 to 2.2).

Similarly, a meta-analysis by Wang et al. [27], including nine RCTs with 7,182 participants, found that long-acting CAB/RPV significantly reduced the risk of treatment discontinuation (risk ratio (RR) = 0.52; 95% CI: 0.38-0.71) and increased patient preference for injectable therapy (odds ratio (OR) = 6.24; 95% CI: 4.88-7.98). A subsequent global meta-analysis by Blanco et al. [28] comprising over 6,000 participants confirmed that CAB/RPV-LA was associated with a significantly lower discontinuation rate (RR = 0.61; 95% CI: 0.47-0.78) and higher satisfaction and adherence compared with standard oral ART.

Across all included studies, more than 92% of patients receiving long-acting cabotegravir-rilpivirine achieved or maintained viral suppression through 48-96 weeks, consistent with pooled estimates reported by Hedima et al. (94.1% vs. 93.6% for oral ART; RR = 1.01; 95% CI: 0.97-1.04) [29]. These findings confirm the virological non-inferiority of long-acting regimens within the populations studied, which predominantly consisted of treatment-experienced, virally suppressed individuals meeting strict eligibility criteria. Importantly, this evidence does not extend to patients initiating ART with high baseline viral loads or to those re-engaging in care with a history of poor adherence, for whom standard oral regimens remain the preferred option [30]. Patient satisfaction, assessed through the HIV treatment satisfaction questionnaire [31], was consistently higher with CAB/RPV-LA, with mean score improvements ranging from +4.1 to +10.4 points across trials [18-22,24,26]. This aligns with findings by Pfau et al. [32], who reported that over 85% of patients preferred injectables due to convenience, reduced stigma, and lower dosing frequency.

Regarding safety, adverse event rates were comparable across treatment arms. ISRs (pain, erythema, induration) occurred in up to 75% of participants but were predominantly mild to moderate and led to treatment discontinuation in <1% of cases [33]. These results are consistent with Orkin et al. and Overton et al., who reported the transient and manageable nature of these reactions [19,21]. Adherence findings were particularly robust in real-world studies. The JABS study demonstrated adherence of 97.2% within the dosing window (±7 days) [26], suggesting that long-acting injectables can enhance continuity in populations with historically poor adherence [34]. Blanco et al. [28] corroborated these results, showing a 48% reduction in treatment discontinuation among socially vulnerable groups.

Limitations

Most trials employed open-label designs, introducing potential subjective bias in self-reported satisfaction and adverse events. Nonetheless, all studies met adequate methodological quality (Jadad ≥3), and no significant inconsistencies were identified under GRADE assessment [17,19-26]. Within the RCTs included in this systematic analysis, resistance development was rare, with no consistent signal of emergent resistance observed across studies. Outside the included trials, isolated cases of NNRTI- and integrase strand transfer inhibitor-associated resistance mutations during cabotegravir-rilpivirine therapy have been reported in the literature [35,36]. However, delayed dosing or missed injections could theoretically increase resistance risk [37], emphasizing the need for active monitoring systems and adherence support.

An important limitation relates to the generalizability of the available evidence. The majority of included studies enrolled treatment-experienced, virologically suppressed individuals without hepatitis B coinfection, which limits extrapolation to patients initiating ART with high baseline viral loads, those re-engaging in care after periods of non-adherence, or individuals requiring ART regimens active against hepatitis B. In addition, study populations were predominantly male, although this distribution may partly reflect the demographic characteristics of HIV cohorts in many real-world settings.

Evidence in adolescents remains limited; although studies such as Moore et al. demonstrated safety and maintained viral suppression in virologically controlled youth, further research is needed before widespread implementation [38,39]. Additionally, mild weight gain, particularly among women, was reported in some of the studies included in this review [22]. Similar weight changes have also been described in external studies not included in the present systematic analysis, potentially reflecting class effects associated with integrase strand transfer inhibitors [40]. Importantly, African trials such as CARES reported efficacy comparable to that observed in high-income countries, supporting feasibility in resource-limited settings, while still acknowledging population- and system-level constraints [22]. As noted by Calleja et al. [41], scaling up CAB/RPV-LA in settings with structural barriers to adherence represents both a challenge and a strategic opportunity for public health optimization.

## Conclusions

The evidence synthesized in this systematic review consistently demonstrates that CAB/RPV-LA is an effective and safe alternative to daily oral ART in adults living with HIV. Across 10 RCTs including more than 5,600 participants and follow-up periods extending up to 240 weeks, CAB/RPV-LA achieved durable virological suppression rates exceeding 90%, confirming its non-inferiority to standard oral regimens. The safety profile was favorable, with adverse events predominantly limited to mild-to-moderate ISRs and treatment discontinuation rates below 1%, supporting the clinical feasibility of this regimen in both controlled trial settings and real-world practice.

Beyond virological efficacy, these findings highlight the added value of CAB/RPV-LA from a patient-centered perspective, as its use was consistently associated with higher treatment satisfaction, improved perceived adherence, and a reduced daily treatment burden. These advantages are particularly relevant for individuals experiencing treatment fatigue, adherence challenges, or stigma related to daily oral therapy. Collectively, the results support the integration of long-acting injectable regimens into long-term HIV management strategies, while underscoring the importance of appropriate patient selection, structured follow-up systems, and further research in underrepresented populations to optimize clinical and public health impact.
